# Ambient Air Pollution and Vision Disorder: A Systematic Review and Meta-Analysis

**DOI:** 10.3390/toxics12030209

**Published:** 2024-03-10

**Authors:** Zhuo Han, Chao Zhao, Yuhua Li, Meng Xiao, Yuewei Yang, Yizhuo Zhao, Chunyu Liu, Juan Liu, Penghui Li

**Affiliations:** 1 School of Environmental Science and Safety Engineering, Tianjin University of Technology, Tianjin 300384, China; hzzzz1pajk@163.com (Z.H.); 18835547676@163.com (M.X.); yyw15133010732@163.com (Y.Y.); zyz@stud.tjut.edu.cn (Y.Z.); liuchunyu981002@163.com (C.L.); 2 Tianjin Key Laboratory of Hazardous Waste Safety Disposal and Recycling Technology, Tianjin 300384, China; 3 School of Chemical Engineering and Technology, Tianjin University, Tianjin 300072, China; zhao_chao@tju.edu.cn; 4 Shandong Provincial Eco-Environment Monitoring Center, Jinan 250100, China; liyuhua01@shandong.cn

**Keywords:** air pollution, PM_2.5_, NO_2_, vision disorder, meta-analysis, odds ratio

## Abstract

The effects of air pollution on physical health are well recognized, with many studies revealing air pollution’s effects on vision disorder, yet no relationship has been established. Therefore, a meta-analysis was carried out in this study to investigate the connection between vision disorder and ambient particles (diameter ≤ 2.5 µm (PM_2.5_), diameter ≤ 10 µm (PM_10_)) and gaseous pollutants (nitrogen dioxide (NO_2_), sulfur dioxide (SO_2_), carbon monoxide (CO), Ozone (O_3_)). Twelve relevant studies published by 26 February 2024 were identified in three databases. A pooled odds ratios (ORs) of 95% confidence intervals (CIs) were obtained using random-effects meta-analysis models. Meta-analysis results revealed that for every 10 µg/m^3^ increase in PM_2.5_ and NO_2_ exposure, a substantially higher incidence of vision disorder was observed (OR = 1.10; 95% CI: 1.01, 1.19; OR = 1.08, 95% CI: 1.00, 1.16). No significant correlation existed between exposure to PM_10_, SO_2_ and CO and vision disorder. However, O_3_ exposure was negatively associated with vision disorder. In addition, subgroup analyses revealed that PM_2.5_ exposure was significantly correlated with the risk of glaucoma and age-related macular degeneration and that children and adolescents were more susceptible to NO_2_ and PM_2.5_ than adults. Overall, exposure to air pollutants, especially PM_2.5_ and NO_2_, may increase the incidence of vision disorder.

## 1. Introduction

Globally, vision disorder has become an issue with serious adverse effects on people’s health and affects people’s opportunities in the workplace and school [1]. Vision disorder refers to a limited ability to visually respond to light and structural stimuli due to lesions in the eye or central visual pathways [2]. The most common clinical symptoms include refractive errors (such as nearsightedness, farsightedness, or astigmatism), glaucoma, cataracts, age-related macular degeneration (AMD), and retinal damage caused by diabetes (diabetic retinopathy). According to the included articles, common and severe vision disorders include glaucoma, cataract and AMD, which were taken as the outcome criteria for vision disorder in this study. Age-related degenerative neuropathy glaucoma is a significant contributor to vision disorder and blindness worldwide [3]. Besides increased intraocular pressure, a sufficient but not necessary causing factor for glaucoma greater exposure to PM_2.5_ has been revealed by studies to be associated with its adverse structural features. Thus, air pollution could be a possible risk factor for glaucoma. Cataracts, opacification of the ocular lens, are major reason of functional vision loss [4]. Air related factors such as ultraviolet light, high temperatures, and lack of oxygen may contribute to the formation of cataracts. AMD is a disorder with a late start that results in lipid-rich extracellular deposits, localized inflammation, and eventually neurodegeneration in the macula, the center of the retina [5]. Glaucoma, cataracts, and AMD share some common pathophysiological mechanisms, including increased inflammation and oxidative stress. Should there be a negative correlation between air pollution and vision disorder, then air pollution may be a novel and potentially modifiable risk factor.

Today, air pollution ranks fifth in terms of risks to public health and is an environmental threat to well-being at the global scale, affecting all populations to some extent [6,7]. The World Health Organization (WHO) lists PM (Particulate Matter), O_3_ (Ozone), NO_2_ (nitrogen dioxide), and SO_2_ (sulfur dioxide) as the four most significant air pollutants [8]. Air pollution has long been a serious environmental concern because it can cause a wide range of health problems at different stages of life [9]. Our eyes are continuously taking in the world around us, also various air pollutants [9,10]. Conjunctivitis and dry eye risks are increased by air pollution, and oxidative stress from air pollutants also affect other eye illnesses, according to previous research [5,11].

Recent epidemiological studies have assessed how air pollution exposure and vision disorders are related [10,12,13]. However, our analysis of the pertinent literature revealed that racial disparities, pollution levels, lifestyle choices, and recognized risk factors for vision disorder, such as age, region (The research regions included in this study include China, South Korea, the United Kingdom and Canada), and gender, may have an impact on the findings [14,15]. According to one research exposure to SO_2_ and CO (carbon monoxide) was positively related to the prevalence of vision disorder in children [13]. Meanwhile, in another study, PM_10_ (inhalable particles), NO_2_, and SO_2_ levels were not associated with cataracts [10]. It follows that these associations are heterogeneous. A summary of the connection between ambient air pollution and vision disorder is thus necessary. To evaluate the state of the art and point the way toward further exploration, a meta-analysis was carried out.

## 2. Materials and Methods

### 2.1. Search Strategy

As of 26 February 2024, two reviewers independently conducted literature searches on the risk of air pollution and vision disorder outcomes in electronic databases, including PubMed, Embase, and Web of Science. The search strategy is based on a combination of vision disorder (visual impairment, visual disorders, ‘disorder, visual’, visual disorder, macropsia, visual impairment, micropsia, vision disability, hemeralopia, metamorphopsia) and ambient air pollutants (air pollutants, atmospheric pollutants, sulfur dioxide, nitrogen dioxide, carbon monoxide, ozone, particulate matter, PM, PM_10_, PM_2.5_, VOCs) keywords. Specific search strategies are provided in Supplementary Table S1.

### 2.2. Inclusion and Exclusion Criteria

All studies were independently reviewed by two investigators (ZH and MX), and a third independent investigator (YY) was called upon to reach a consensus in case of any disputes. The following are the inclusion requirements: (1) original research; (2) population-based studies; (3) studies that observe something, such as a cohort, a case–control study, or a cross-sectional study; (4) exposure to particle and gaseous contaminants in the air, including PM_1_, PM_2.5_, PM_10_, NO_2_, SO_2_, CO and O_3_; (5) studies providing ORs, relative risk (RR), or hazard ratios (HRs) with 95% CIs for the visual impairment outcomes associated with any air pollutants; and (6) articles in English.

The following were the exclusion requirements: (1) studies in which no data can be retrieved; (2) studies involving animal experiments; (3) studies of poor quality; (4) comments, letters, responses to review articles, and meta-analyses.

### 2.3. Quality Assessment

The following techniques were employed to assess the quality of the literature by the study types of the included articles: (1) Cross-sectional study statistics assessment and review instrument meta-analysis by the Joanna Briggs Institute (JBI) (Table S2) [16]; (2) 9-star Newcastle–Ottawa Scale (NOS) (Table S3) for cohort studies and case–control studies [17]. In our study, the JBI scale contained 10 items on a scale of 0 to 20, each rated on a scale of 2 (detailed, comprehensive, and correctly described); 1 (mentioned but not described in detail); and 0 (not met). Studies are categorized as “high quality” if they receive a JBI score of at least 16, as opposed to “low quality” otherwise [16]. The NOS scale had a total score that ranged from 0 to 9, and the study’s quality was determined by its selection (0–4 points), comparability (0–2 points), and outcome (0–3 points) quality factors. The study quality was rated on a scale of 0–3 as low, 4–6 as medium, and 7–9 as high [17]. Supplementary Tables S4 and S5 of the Supplementary Materials detailed the grading system.

### 2.4. Data Extraction

Two investigators retrieved data from all included studies separately and in a defined way, and third investigator resolved disagreements through discussion. For every eligible study, we extracted the initial author’s name, publication year, study site, time frame, study design, sample size, population characteristics, pollution characteristics, assessment method, adjusted variables, outcome definition, time of assessment, type of outcome and subgroup analysis results, effect size (OR, RR or HR, 95% CI) of the correlation between air pollutants and vision disorder.

We transformed all air pollutant measurement units to µg/m^3^ to standardize impact sizes: (1) 1 ppm = 1000 ppb, 1 mg/m^3^ = 1000 µg/m^3^; (2) NO_2_: 1 ppb = 46/22.4 µg/m^3^; (3) O_3_: 1 ppb = 48/22.4 µg/m^3^; (4) SO_2_: 1 ppb = 64/22.4 µg/m^3^; (5) CO: 1 ppb = 28/22.4 µg/m^3^ [15]. After that, all effect estimates were combined for a 10 µg/m^3^ rise in pollutant concentration. The following formulas were used to transform the standard risks for each investigation [18]:

OR_(standardized)_ = OR_(original)_^Increment(10)/Increment(original)^

### 2.5. Statistical Analysis

Statistical analysis was carried out using Stata 17.0. ORs and their 95% CIs, which were mostly used in studies with various designs, populations, and follow-up times, were used to present pooled data. Other effect sizes were converted into ORs. Forest plots and standard cut-offs for *I*^2^ statistics were used to assess heterogeneity across studies. Heterogeneity was ranked as low (*I*^2^ ≤ 25%), medium (25% < *I*^2^ < 75%), and high (*I*^2^ ≥ 75%) at those percentages. Subgroup and sensitivity analyses were done to look into the causes of heterogeneity. When the values of *I*^2^ were greater than 50%, the random-effects inverse-variance model was used to compute the combined estimates. Moreover, statistical significance was assumed when the *p*-value of a two-tailed test was less than 0.05 [19].

## 3. Result

### 3.1. Study Results

**Figure 1:**
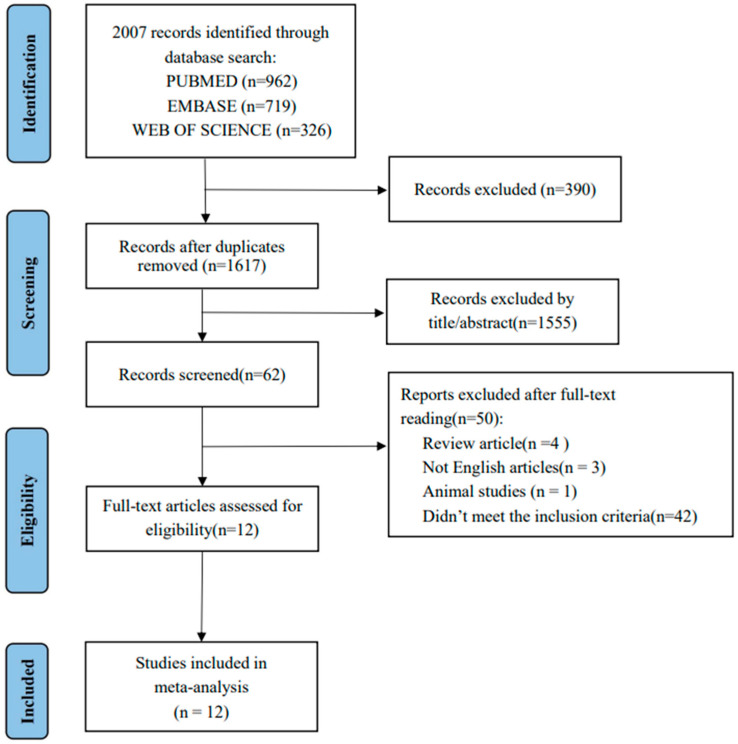
Flow chart of the literature search and selection for meta-analysis.

Using three electronic databases, we screened 2007 articles in total and eliminated 390 duplicates. The titles and abstracts of the remaining 1617 articles were preliminarily screened, of which 1555 were excluded after the initial screening, and a total of 62 articles were screened for full-text reading. Considering the inclusion and exclusion standards, 50 studies were excluded, of which four were reviews, one was an animal study, three were non-English articles, and 42 did not meet the inclusion criteria (Figure 1). Twelve studies [3,5,10,12,13,20,21,22,23,24,25,26] evaluating the relationship between air pollutant exposure and the risk of vision disorder outcomes were included in this meta-analysis.

### 3.2. Study Characteristics

The characteristics of the included studies are summarized in Table 1. Twelve studies were published between 2018 and 2022, with the studies covering the period from 2000 to 2021. Seven were cross-sectional, three were cohort, and two were case-crossover studies. The studies were carried out in four nations: China (n = 6), South Korea (n = 3), the United Kingdom (n = 2), and Canada (n = 1), and they involved a large number of participants, ranging from 3225 to 340,313. Our review included the following number of studies on various pollutants: PM_10_ (n = 6), PM_2.5_ (n = 10), PM_1_ (n = 1), NO_2_ (n = 7), SO_2_ (n = 4), CO (n = 5), and O_3_ (n = 3). Regarding quality assessment, twelve studies met the criteria for good quality (Supplementary Tables S3 and S5). Most included studies estimated the correlations between air pollution and vision disorder outcomes by multivariable multiple logistic regression and multiple cox proportional hazards regression models, and evaluated OR, RR or HR with 95% CIs for each air pollutant selected.

### 3.3. The Association between Environmental Air Pollutants Exposure and Vision Disorder

Twelve studies looked into the connection between exposure to air pollution and vision disorder; nine reported ORs with 95% CIs, two supplied HRs with 95% CIs, and one reported RRs with 95% CIs. We estimated the pooled ORs for vision disorders (cataract, glaucoma, AMD and visual impairment) associated with each air pollutant. Three, five, four and two studies assessed the relationship between air pollutant exposure and cataract, glaucoma, AMD and visual impairment, respectively. The correlations of vision disorder with exposure to PM_10_, PM_2.5_, PM_1_, SO_2_, NO_2_, O_3_ and CO were reported in studies (Table 2 and Table 3).

**Figure 2:**
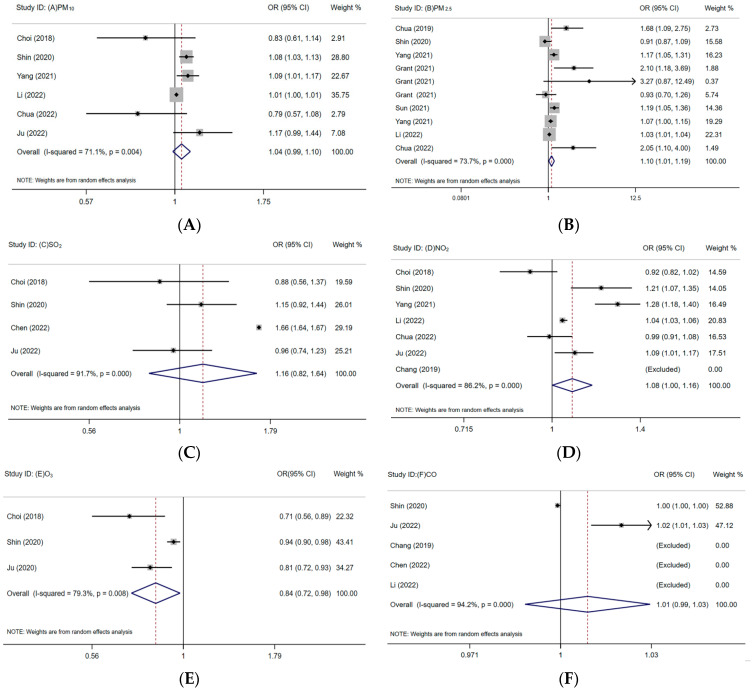
Associations of PM_10_ (**A**), PM_2.5_ (**B**), SO_2_ (**C**), NO_2_ (**D**), O_3_ (**E**) and CO (**F**) with vision disorder. (1. A solid line perpendicular to the X-axis and with a horizontal axis of 1 is an invalid line; 2. Multiple line segments parallel to the horizontal axis represent the 95% CI of each included study, and black dots represent the OR value of each study; 3. Arrow: The 95% CI of the OR value in this study exceeds the display range of the graph; 4. The diamond represents the summary results of multiple studies, where the dashed line perpendicular to the X-axis and passing through the center of the diamond represents the merged effect value, and the width of the diamond represents 95% CI of the merged results; 5. The area of gray squares represents weight, and the larger the weight, the larger the square area) [3,5,10,12,13,20,21,22,23,24,25,26].

The combined results suggested that air pollutants could boost the likelihood of having a vision disorder, with the combined OR (95% CI) of 1.10 (1.01–1.19) and 1.08 (1.00–1.16) per 10 µg/m^3^ increment in exposure to PM_2.5_ and NO_2_, respectively (Figure 2). But the results showed that PM_10_ (OR = 1.04, 95% CI: 0.99, 1.10; *I*^2^ = 71.1%, *p* = 0.004) (Figure 2A), SO_2_ (OR = 1.16, 95% CI: 0.82, 1.64; *I*^2^ = 97.1%, *p* = 0.000) (Figure 2C) and CO (OR = 1.01, 95% CI: 0.99, 1.03; *I*^2^ = 94.2%, *p* = 0.000) (Figure 2F) were not significantly associated with vision disorder. The pooled OR from all studies between O_3_ exposure and vision disorder was 0.84 (95% CI: 0.72, 0.98) with significant heterogeneity (*I*^2^ = 79.3%, *p* = 0.008) (Figure 2E). To further investigate the causes of this variation, we performed a meta-regression and a subgroup analysis.

### 3.4. Subgroup Analyses

**Figure 3:**
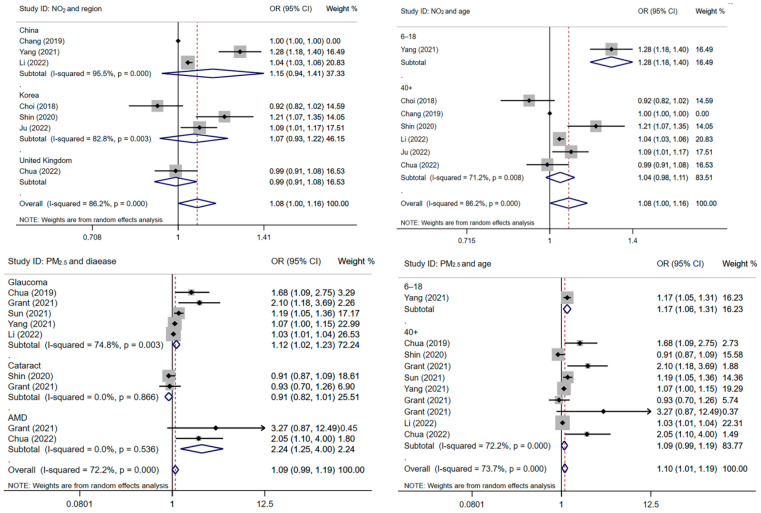
The effect of NO_2_ and PM_2.5_ on vision disorder, stratified by region, disease and age. (1. A solid line perpendicular to the X-axis and with a horizontal axis of 1 is an invalid line; 2. Multiple line segments parallel to the horizontal axis represent the 95% CI of each included study, and black dots represent the OR value of each study; 3. Arrow: The 95% CI of the OR value in this study exceeds the display range of the graph; 4. The diamond represents the summary results of multiple studies, where the dashed line perpendicular to the X-axis and passing through the center of the diamond represents the merged effect value, and the width of the diamond represents 95% CI of the merged results; 5. The area of gray squares represents weight, and the larger the weight, the larger the square area) [3,5,10,12,20,21,22,23,24,25,26].

We first performed a subgroup study based on how NO_2_ affected different regions (China, Korea and the United Kingdom) and age (6–18, 40+) (Figure 3). Secondly, we conducted a subgroup analysis of age and different common diseases of vision disorder according to PM_2.5_ (Figure 3). The findings demonstrated that there was no statistically strong correlation between elevated NO_2_ concentrations and the risk of vision disorder in China, Korea, and the United Kingdom, with combined ORs of 1.15 (95% CI: 0.94, 1.41), 1.07 (95% CI: 0.93, 1.22) and 0.99 (95% CI: 0.91, 1.08). Additionally, the impact of PM_2.5_ on various glaucoma, cataract, and AMD diseases was revealed by subgroup analysis. We can obtain the combined effect of studies with the risk of glaucoma (OR = 1.12, 95% CI: 1.02, 1.23), cataract (OR = 0.91, 95% CI: 0.82, 1.01), AMD (OR = 2.24, 95% CI: 1.25, 4.00). It can be concluded that PM_2.5_ is positively correlated with glaucoma and AMD, while there is no significant correlation between cataract. Finally, subgroup analysis by age level showed that PM_2.5_ (OR = 1.17, 95% CI: 1.06, 1.31) and NO_2_ (OR = 1.28, 95% CI: 1.18, 1.40) were positively correlated with vision disorder in children and adolescents, but not significantly correlated with adults over 40 years of age.

### 3.5. Sensitivity Analysis and Publication Bias

The stability of the pooled data was evaluated using the one-study deletion sensitivity analysis, which involved repeatedly combining estimations and removing one study at a time. Supplementary Figures S1–S6 of the results reveal that the majority of the pollutant consolidation results are steady. We also evaluated the potential publication bias with funnel plots (Supplementary Figures S7–S12) when 10 or more studies were included [27]. The funnel plot can be used to evaluate publication bias visually. Additionally, we used an Egger test to evaluate publication bias. Egger’s tests suggested that there occurred no significant publication bias in PM_10_, NO_2_, and O_3_ exposure on vision disorder (*p*-value of the Egger’s test > 0.05), while PM_2.5_ and SO_2_ exposure had publication bias on vision disorder (*p* < 0.05) (Supplementary Table S6).

## 4. Discussion

To our knowledge, this study is the first to thoroughly evaluate the link between exposure to air pollution and vision disorder. Although a similar systematic review has been conducted, it compares the age-related burden of eye disease in adults exposed to ambient air pollutants [28]. Our study differs from previous studies in that we included literature on children and adolescents in addition to adults, and estimated the combined effect of ambient air pollution on their vision disorders. Twelve studies were included after being retrieved from the system. After pooling the effect estimates from the 12 studies, it is found that the increased concentration of PM_2.5_ and NO_2_ may increase the incidence of vision disorder, whereas no significant association between PM_10_, SO_2_, CO and vision disorder was observed. In addition, earlier research indicated that exposure to O_3_ was positively correlated with the risk of vision disorder. However, this investigation found that O_3_ was negatively associated with vision disorder. The explanation for the results of this meta-analysis might be due to the inclusion of different studies and study designs in the included articles. Therefore, further research is needed to understand this relationship fully.

Exposure to air pollutants raises the chance of vision disorder, and previous research has shown that air pollution may directly irritate the eyes, especially in the cornea and conjunctiva [9,29,30]. This study showed that exposure to PM_2.5_ and NO_2_ was positively correlated with vision disorder. Previous researchers used fluorescent PM_2.5_ tracers to understand how PM_2.5_ enters the eye. They discovered that particles of a size between 10 and 500 nm entered the anterior chamber via the cornea and were mostly deposited in the outflow tissue, with the majority of the particles staying in the ciliary body. This study suggests that PM_2.5_ exposure may impact the cornea’s connective tissue biomechanical capabilities [3,20]. The positive correlation between NO_2_ and vision disorder may be explained by the slow hydrolysis of NO_2_ into nitrous and nitric acid after respiratory inhalation, which causes lipid peroxidation and oxidative stress [5]. In addition, since the retina is a component of the central nervous system, it makes biological sense that it may be susceptible to NO_2_ poisoning [5].

In a prior study, higher ozone concentrations were linked to dry eye disease [31], and other research demonstrated that ozone exposure causes ocular surface degradation and an inflammatory state [11]. Therefore, we expected that there might be a positive association between ozone and vision disorder. Contrary to our expectations, this study found a negative correlation between ozone and vision disorder. A possible explanation is that ozone is a well-known polar molecule, that may not penetrate the cornea easily [10]. Therefore, the lens may be immune to the oxidative damage caused by ozone [10]. Another possible explanation is that exposure to ultraviolet (UV) radiation is the major cause of oxidative stress and the most significant risk factor for cataract development [32]. Less UV radiation may reach the surface and enter the eye due to the stratospheric ozone layer’s filtering of UV rays [10]. It also reduces the vision disorder caused by oxidative stress caused by ultraviolet light.

Subgroup analyses were conducted to investigate possible causes of heterogeneity in the meta-analysis. The findings showed that study region, disease and age are the primary causes. Subgroup analysis showed no significant correlation between NO_2_ exposure and vision disorder in China, South Korea and the United Kingdom. Possible explanations are the limited number of studies and potential sources of heterogeneity such as demographics, participant characteristics, sample size, and regional environmental air pollution monitoring. In addition, subgroup analysis of diseases found that PM_2.5_ exposure was significantly correlated with the risk of glaucoma and AMD. With glaucoma and AMD being multifactorial neurodegenerative illnesses that may result in the death of retinal ganglion cells and visual field abnormalities, there is growing evidence that air pollution may have a role in the development of neurodegenerative disorders [33,34]. Therefore, this may be why PM_2.5_ in the subgroup analysis is positively correlates with glaucoma and AMD. Cataracts develop from many factors: metabolic disorders, dietary deficiency, or environmental stressors, including severe cold or heat, radiation, metal ions, and toxins [10,12]. The subgroup analysis results show that the correlation between PM_2.5_ and the incidence of cataracts is insignificant, possibly because PM_2.5_ in the air is not a crucial factor affecting cataract occurrence among the above-mentioned factors. Finally, we found that air pollution affects children and adolescents more than adults due to their exposure level and physiological characteristics. Due to their increased ventilation rates and frequent outside activity, children and adolescents may be exposed to air contaminants more often [35]. In addition, the bodies of children and adolescents are still growing and their immune systems are still underdeveloped, which makes them less resistant to air pollution than adults.

No gender-based subgroup analyses were carried out in this study due to data limitations, although recent research has indicated gender variations. The gender-specific effects can be attributable to socially derived air pollution exposures. In addition, there are also gender differences in the human body’s gas-blood barrier permeability, particle deposition, and gas absorption [36]. For example, Studies have shown that many human organs may be affected by indoor air pollution, where the eyes are directly exposed to emissions from the burning of solid fuels, including high levels of fine PM_2.5_ and CO [37]. The higher association between cataracts in women than men, considering the mixed effects of women’s exposure to indoor cooking fuels and outdoor activities on cataracts [38]. On the contrary, for children, boys spend more time outside and are more active than girls, which exposes them to more air pollution and may make them more vulnerable to its effects [23].

Several mechanisms have been suggested to explain these findings. Studies have shown that for cataracts, oxidative stress of reactive oxygen species and reactive nitrogen (ROS/RNS) is considered the main formation mechanism [39]. Oxidative stress caused by air pollution is the stressor inducing cataracts, which may harm the membrane cavity and secreted proteins [10]. The integrity of the cornea’s barrier may be altered by PM and NO_x_, which may also encourage the creation of ROS and cause inflammation of the retina and ocular surface [9,40]. As was previously discussed, atmospheric particulate matter and NO_2_ may produce ROS/RNS and trigger oxidative damage to a wide range of biomolecules [40]. Therefore, oxidative stress and inflammation are mechanisms that explain the effect of air pollutants on the occurrence of eye diseases. The corresponding reduction of air pollutants may affect the pathogenesis of vision disorder and thus reduce the incidence of vision disorder.

The results suggested that air pollution is correlated with vision disorder, in which PM_2.5_ and NO_2_ exposure may be positively correlated with vision disorder-related risk, PM_10_, SO_2_ and CO exposure have no significant effect on vision disorder, and O_3_ exposure is negatively associated with vision disorder. The association varied by region, disease and age. PM_2.5_ exposure was significantly correlated with the risk of glaucoma and AMD, but with cataracts not significant. Children and adolescents are more vulnerable to the impacts of air pollution than adults. In addition, the OR value can reflect the strength of the association between air pollutants and vision disorder. In Figure 2, the middle vertical line is the invalid line, OR = 1. When the combined OR value is on the right side of the invalid line, it means that the study factor (air pollutant) and the outcome (vision disorder) are in a positive relationship, and the farther away from the invalid line, the OR value is greater than 1, and the greater the correlation strength. Therefore, PM_2.5_ is more strongly associated with vision disorder than NO_2_.

This systematic review and meta-analysis have some limitations. First, the cross-sectional nature does not determine causality between studies [23]. Second, the dearth of research made it impossible to analyze the potential sources of heterogeneity thoroughly. This suggests that variations in population factors, participant characteristics, sample size, and geographical location may be at play. Third, considering heterogeneity and the small amount of studies for every air contaminant, care should be exercised when interpreting the findings. Fourth, we classified the diseases associated with vision disorders. However, in the included articles, only cataracts, glaucoma, age-related macular degeneration and visual impairment were studied as outcomes, and no mention was made of hyperopia, myopia, night blindness and deformities. Above all, misclassification of exposures was unavoidable since data from monitoring stations was utilized in practically all research. In addition, we did not analyze PM_1_ based to the limitations of the available literature.

Based on previous studies, to better understand the relationship between air pollutants and vision disorder, here are some ideas on where to take subsequent studies: (1) Additional large-scale, long-term cohort studies are required for a more accurate and trustworthy evaluation. (2) More careful monitoring of exposure levels. (3) More research is needed to determine the effects of air pollution on vision disorders in terms of genetics, demographics, social variables, and behaviors. (4) Multiple pollutant interactions and their consequences on vision disorder have yet to be quantified.

## 5. Conclusions

In conclusion, ambient air pollution may contribute to vision disorder. PM_2.5_ and NO_2_ are air pollutants correlated with an increased risk of vision disorder. The correlation varied by region, disease and age. The results indicate that policymakers might anticipate the likelihood of vision disorder due to air pollution and adopt targeted preventative actions in advance. In the future, more relevant research is necessary to provide a more accurate and reliable assessment.

## Figures and Tables

**Table 1:** Characteristics of studies included in the systematic review and meta-analysis.

Study	Location	Data Period	Design	Sample Size	Age	Exposure Pollutant(s)	Statistical Model	Outcome Type	Quality
Choi et al., 2018 [10]	Republic of Korea	2006–2012	Cross-sectional study	18,622	40+	O_3_, NO_2_, SO_2_, PM_10_	Multiple logistic regression analyses	Cataract	17/20
Chua et al., 2019 [3]	United Kingdom	2006–2010	Cross-sectional study	111,370	40–69	PM_2.5_	Multiple logistic regression analyses	Glaucoma	18/20
Chang et al., 2019 [5]	China-Taiwan	2000–2010	Longitudinal population-based study	39,819	50+	NO_2_, CO	Multiple Cox proportional hazards regression	AMD	6/9
Shin et al., 2020 [21]	Republic of Korea	2002–2015	Longitudinal population-based study	115,728	50+	PM_2.5_, PM_10_, NO_2_, CO, SO_2_, O_3_	Multiple Cox proportional hazards regression	Cataract	9/9
Yang et al., 2021 [23]	China	2010–2013	Cross-sectional study	61,995	6–18	PM_1_, PM_2.5_, PM_10_, NO_2_	SAS PROC SURVEYLOGISTIC, SAS PROC SURVEYREG	Visual impairment	16/20
Grant et al., 2021 [20]	Canada	2011–2015	Cross-sectional population-based study	30,097	45–85	PM_2.5_, O_3_, SO_2_, NO_2_	Multiple logistic regression analyses	AMD, Cataract, Glaucoma, Visual impairment	19/20
Sun et al., 2021 [22]	China-Taiwan	2008–2013	Nested case–control study	3225	65+	PM_2.5_	Multiple logistic regression analyses	Glaucoma	6/9
Yang et al., 2021 [24]	China	2000–2016	Cross-sectional study	33,701	40+	PM_2.5_	Multiple logistic regression analyses	Glaucoma	16/20
Chen et al., 2022 [13]	China	2005–2018	Longitudinal, two-center cohort study	340,313	SD: 11.30 (±2.64)	SO_2_, CO	Multiple Cox proportional hazards regression	Visual impairment	8/9
Li et al., 2022 [26]	China	2015–2021	Case-crossover study	14,385	SD: 56.79 (±15.33)	PM_2.5_, PM_10_, NO_2_, CO	Conditional logistic regression model	Glaucoma	7/9
Chua et al., 2022 [12]	United Kingdom	2006–2010	Cross-sectional study	115,954	40–69	PM_2.5_, PM_10_, NO_2_	Multiple logistic regression analyses	AMD	17/20
Ju et al., 2022 [25]	Republic of Korea	2008–2012	Cross-sectional study	15,115	40+	NO_2_, CO, O_3_	Survey-logistic regression models	AMD	16/20

Abbreviations: PM_1_: particle with aerodynamic diameter ≤ 1 µm; SD: The mean age.

**Table 2:** Summary effects and 95% confidence intervals for vision disorder associated with PM.

Air Pollutant	Author (Year)	Outcome Type	Incremental Scale	Original OR/HR	Transformed OR
PM_10_	Choi et al. (2018) [10]	Cataract	5 µg/m^3^	OR: 0.91 (95% CI, 0.78–1.07)	OR: 0.83 (95% CI, 0.61–1.14)
	Shin et al. (2020) [21]	Cataract	IQR: 9.1 µg/m^3^	HR: 1.069 (95% CI, 1.025–1.115)	OR: 1.076 (95% CI, 1.028–1.127)
	Yang et al. (2021) [23]	Visual impairment	IQR: 16.11 µg/m^3^	OR: 1.142 (95% CI, 1.019–1.281)	OR: 1.086 (95% CI, 1.012–1.166)
	Li et al. (2022) [26]	Glaucoma	IQR: 35 µg/m^3^	OR: 1.03 (95% CI, 1.01–1.05)	OR: 1.01 (95% CI, 1.00–1.01)
	Chua et al. (2022) [12]	AMD	IQR: 2.67 µg/m^3^	OR: 0.94 (95% CI, 0.86–1.02)	OR: 0.79 (95% CI, 0.57–1.08)
	Ju et al. (2022) [25]	AMD	IQR: 8 µg/m^3^	OR: 1.13 (95% CI, 0.99–1.34)	OR: 1.17 (95% CI, 0.99–1.44)
PM_2.5_	Chua et al. (2019) [3]	Glaucoma	IQR: 1.12 µg/m^3^	OR: 1.06 (95% CI, 1.01–1.12)	OR: 1.68 (95% CI, 1.09–2.75)
	Shin et al. (2020) [21]	Cataract	IQR: 7.0 µg/m^3^	HR: 0.905 (95% CI, 0.772–1.062)	OR: 0.905 (95% CI, 0.867–1.090)
	Yang et al. (2021) [23]	Visual impairment	14.79 µg/m^3^	OR: 1.267 (95% CI, 1.082–1.484)	OR: 1.174 (95% CI, 1.055–1.306)
	Grant et al. (2021) [20]	Glaucoma	IQR: 2.9 µg/m^3^	OR: 1.24 (95% CI, 1.05–1.46)	OR: 2.10 (95% CI, 1.18–3.69)
	Grant et al. (2021) [20]	AMD (with visual impairment)	IQR: 2.9 µg/m^3^	OR: 1.41 (95% CI, 0.96–2.08)	OR:3.27 (95% CI, 0.87–12.49)
	Grant et al. (2021) [20]	Cataract	IQR: 2.9 µg/m^3^	OR: 0.98 (95% CI, 0.90–1.07)	OR: 0.93 (95% CI, 0.70–1.26
	Sun et al. (2021) [22]	Glaucoma	10 µg/m^3^	OR: 1.19 (95% CI, 1.05–1.36)	OR: 1.19 (95% CI, 1.05–1.36)
	Yang et al. (2021) [24]	Glaucoma	10 µg/m^3^	OR: 1.07 (95% CI, 1.00–1.15)	OR: 1.07 (95% CI, 1.00–1.15)
	Li et al. (2022) [26]	Glaucoma	IQR: 26 µg/m^3^	OR: 1.07 (95% CI, 1.03–1.11)	OR: 1.03(95% CI, 1.01–1.04)
	Chua et al. (2022) [12]	AMD	IQR: 1.07 µg/m^3^	OR: 1.08 (95% CI, 1.01–1.16)	OR: 2.05(95% CI, 1.10–4.00)
PM_1_	Yang et al. (2021) [23]	Visual impairment	10.24 µg/m^3^	OR: 1.133 (95% CI, 1.035–1.240)	OR: 1.130 (95% CI, 1.034–1.234)

**Table 3:** Summary effects and 95% confidence intervals for vision disorder associated with SO_2_, NO_2_, O_3_, CO.

Air Pollutant	Author (Year)	Outcome Type	Incremental Scale	Original OR/HR	Transformed OR
SO_2_	Choi et al. (2018) [10]	Cataract	0.003 ppm	OR: 0.90 (95% CI, 0.62–1.30)	OR: 0.88 (95% CI, 0.56–1.37)
	Shin et al. (2020) [21]	Cataract	IQR: 0.7 ppb	HR: 1.027 (95% CI, 0.984–1.073)	OR: 1.147 (95% CI, 0.920–1.439)
	Chen et al. (2022) [13]	Visual impairment	IQR: 16.16 µg/m^3^	RR: 2.26 (95% CI, 2.22–2.29)	OR: 1.66 (95% CI, 1.64–1.67)
	Ju et al. (2022) [25]	AMD	IOR: 1 ppb	OR: 0.99 (95% CI, 0.92–1.06)	OR: 0.96 (95% CI, 0.74–1.23)
NO_2_	Choi et al. (2018) [10]	Cataract	0.003 ppm	OR: 0.93 (95% CI, 0.85–1.02)	OR: 0.92 (95% CI, 0.82–1.02)
	Chang et al. (2019) [5]	AMD	IQR: 9825.5 ppb	HR: 1.91 (95% CI, 1.64–2.23)	OR: 1.00 (95% CI, 1.00–1.00)
	Shin et al. (2020) [21]	Cataract	IQR: 2.1 ppb	HR: 1.080 (95% CI, 1.030–1.133)	OR: 1.205 (95% CI, 1.074–1.354)
	Yang et al. (2021) [23]	Visual impairment	9.78 µg/m^3^	OR: 1.276 (95% CI, 1.173–1.388)	OR: 1.283 (95% CI, 1.177–1.398)
	Li et al. (2022) [26]	Glaucoma	IQR: 27 µg/m^3^	OR: 1.12 (95% CI, 1.08–1.17)	OR: 1.04 (95% CI, 1.03–1.06)
	Chua et al. (2022) [12]	Glaucoma	10 µg/m^3^	OR: 0.99 (95% CI, 0.91–1.08)	OR: 0.99 (95% CI, 0.91–1.08)
	Ju et al. (2022) [25]	AMD	IQR: 12 ppb	OR:1.24 (95% CI, 1.05–1.46)	OR: 1.09 (95% CI, 1.01–1.17)
O_3_	Choi et al. (2018) [10]	Cataract	0.003 ppm	OR: 0.80 (95% CI, 0.69–0.93)	OR: 0.71 (95% CI, 0.56–0.89)
	Shin et al. (2020) [21]	Cataract	IQR: 5.4 ppb	HR: 0.931 (95% CI, 0.888–0.977)	OR: 0.940 (95% CI, 0.902–0.980)
	Ju et al. (2022) [25]	AMD	IQR: 5 ppb	OR: 0.80 (95% CI, 0.70–0.92)	OR: 0.81 (95% CI, 0.72–0.93)
CO	Chang et al. (2019) [5]	AMD	IQR: 297.1 ppm	HR: 1.84 (95% CI, 1.57–2.15)	OR: 1.00 (95% CI, 1.00–1.00)
	Shin et al. (2020) [21]	Cataract	11 ppm	HR: 0.991 (95% CI, 0.949–1.035)	OR: 0.999 (95% CI, 0.999–1.000)
	Chen et al. (2022) [13]	Visual impairment	1.28 mg/m^3^	RR: 2.30 (95% CI, 2.26–2.35)	OR: 1.01 (95% CI, 1.01–1.01)
	Li et al. (2022) [26]	Glaucoma	IQR: 0.5 mg/m^3^	OR: 1.04 (95% CI, 1.01–1.07)	OR: 1.00 (95% CI, 1.00–1.00)
	Ju et al. (2022) [25]	AMD	IQR: 100 ppb	OR: 1.22 (95% CI, 1.09–1.38)	OR: 1.02 (95% CI, 1.01–1.03)

## Data Availability

The original data presented in the study are included in the article/Supplementary Materials; further inquiries can be directed to the corresponding authors.

## Supplementary Materials

The following supporting information can be downloaded at: https://www.mdpi.com/article/10.3390/toxics12030209/s1, Table S1: Literature search terms; Table S2: JBI evaluation criteria for cross-sectional studies; Table S3: NOS evaluation criteria for cohort studies and case-control studiesQuality; Table S4: assessment for the cross-sectional studies using JBI; Table S5: Quality assessment for the cohort studies and case-control studies using NOS; Table S6: Egger’s test for publication bias of studies exploring exposure to different pollutants and vision disorder; Figure S1: Sensitivity analysis results of PM_10_; Figure S2: Sensitivity analysis results of PM_2.5_; Figure S3: Sensitivity analysis results of SO_2_; Figure S4: Sensitivity analysis results of NO_2_; Figure S5: Sensitivity analysis results of O_3_; Figure S6: Sensitivity analysis results of CO; Figure S7: Funnel plot for publication bias of studies exploring exposure to PM_10_ and vision disorder; Figure S8: Funnel plot for publication bias of studies exploring exposure to PM_2.5_ and vision disorder; Figure S9: Funnel plot for publication bias of studies exploring exposure to SO_2_ and vision disorder; Figure S10: Funnel plot for publication bias of studies exploring exposure to NO_2_ and vision disorder; Figure S11: Funnel plot for publication bias of studies exploring exposure to O_3_ and vision disorder; Figure S12: Funnel plot for publication bias of studies exploring exposure to CO and vision disorder.
