# Supplementary material for: Ambient Air Pollution and Vision Disorder: A Systematic Review and Meta-Analysis
Source: Toxics. 2024 Mar 10;12(3):209. doi: 10.3390/toxics12030209 (PMC10975782; doi:10.3390/toxics12030209)
Supplement: Supplementary file 1 [file toxics-12-00209-s001.zip › toxics-2875674-supplementary.pdf]

Supplementary table and figure legends

Supplementary Table S1: Literature search terms.

Supplementary Table S2: JBI(Joanna Briggs Institute) evaluation criteria for cross-sectional studies.

Supplementary Table S3: Quality assessment for the cross-sectional studies using JBI.

Supplementary Table S4: NOS(Newcastle-Ottawa Scale) evaluation criteria for cohort studies and case-control studies.

Supplementary Table S5: Quality assessment for the cohort studies and case-control studies using NOS.

Supplementary Table S6: Egger's test for publication bias of studies exploring exposure to different pollutants and vision disorder.

Supplementary Figure S1: Sensitivity analysis results of PM_{10}.

Supplementary Figure S2: Sensitivity analysis results of $PM_{2.5}$.

Supplementary Figure S3: Sensitivity analysis results of SO_2.

Supplementary Figure S4: Sensitivity analysis results of NO_2.

Supplementary Figure S5: Sensitivity analysis results of O_3.

Supplementary Figure S6: Sensitivity analysis results of CO.

Supplementary Figure S7: Funnel plot for publication bias of studies exploring exposure to PM_{10} and vision disorder.

Supplementary Figure S8: Funnel plot for publication bias of studies exploring exposure to $PM_{2.5}$ and vision disorder.

Supplementary Figure S9: Funnel plot for publication bias of studies exploring exposure to SO_2 and vision disorder.

Supplementary Figure S10: Funnel plot for publication bias of studies exploring exposure to NO_2 and vision disorder.

Supplementary Figure S11: Funnel plot for publication bias of studies exploring exposure to O_3 and vision disorder.

Supplementary Figure S12: Funnel plot for publication bias of studies exploring exposure to CO and vision disorder.

Supplementary Table S1: Literature search terms

Database	Search Strategies	Results
Pubmed	("Vision disorder" OR "Visual Disorders" OR "Disorder, Visual" OR "Visual Disorder" OR Macropsia OR "Visual Impairment" OR Micropsia OR "Vision Disability" OR Hemeralopia OR Metamorphopsia AND "Air Pollutants" OR "Atmospheric pollutants" OR "Ambient air pollution" OR "SO2" OR "sulfur dioxide" OR "NOx" OR "nitrogen dioxide" OR "carbon monoxide" OR "CO" OR ozone OR O3 OR "particulate matter" OR "PM" OR "PM10" OR "PM2.5" OR "VOCs")	885 records

Embase	#1'Vision disorder' OR 'Visual Disorders' OR 'Visual Disorder' OR Macropsia OR 'Visual Impairment' OR Micropsia OR 'Vision Disability' OR 'Hemeralopia ' OR 'Metamorphopsia ' AND #2'air pollutants' OR 'atmospheric pollutants' OR 'ambient air pollution' OR 'SO2' OR 'sulfur dioxide' OR 'NOx ' OR 'nitrogen dioxide' OR 'carbon monoxide' OR 'CO' OR 'ozone' OR 'O3' OR 'particulate matter' OR 'PM' OR 'PM10 ' OR 'PM2.5 ' OR 'VOCs'	520 records
Web of Science	TS=("air pollutants" or "Environmental Air Pollutants" or "ozone" or "sulfur dioxide" or "nitrogen dioxide" or "carbon monoxide" or "particulate matter" or "vocs" or "PM" or "PM10" or "PM2.5" or "ambient particulate") AND TS=("vision disorder" or "Visual Disorders" or "Disorder, Visual" or "visual Disorder" or "Macropsia" or "visual lmpairment" or "Micropsia" or "vision Disability" or "Hemeralopia" or "Metamorphopsia")	313 records

Supplementary Table S2: JBI(Joanna Briggs Institute) evaluation criteria for cross-sectional studies.

Question	Items	Evaluation criteria (0-2 points)
**Question 1**	Is the purpose of the study clear? Is the question based on sufficient evidence?	0 point: does not meet the requirements;<br>1 point: mentioned but not described in detail;<br>2 points: detailed, comprehensive and correct description.
**Question 2**	How was the study population selected (whether the subjects were randomly selected and whether stratified sampling was used to improve the representativeness of the sample)?	
**Question 3**	Are sample inclusion and exclusion criteria clearly described?	
**Question 4**	Are sample characteristics clearly described?	
**Question 5**	Are the tools used for data collection reliable and valid (if investigators are used, how reproducible are the results)?	
**Question 6**	What are the measures to verify the authenticity of the data?	
**Question 7**	Are ethical issues taken into account?	
**Question 8**	Are the statistical methods correct?	
**Question 9**	Are the findings properly and accurately stated (are the results and inferences distinguished, and are the results	

Question	Items	Evaluation criteria (0-2 points)
	faithful to the data and not the inferences)?	
**Question 10**	Is the value of the research clearly stated?	

Supplementary Table S3: NOS(Newcastle-Ottawa Scale) evaluation criteria for cohort studies and case-control studies.

Cohort study	Study population selection (0-4 points)	Representativeness of the exposed cohort(1 point)	really represents the characteristics of the exposed group in the population ★
			to some extent, it represents the characteristics of the exposed group in the population★
			selected group of users e.g. nurses, volunteers
			no description of the derivation of the cohort
		Selection of the non-exposed cohort(1 point)	from the same population as the exposed group★
			from a different population than the exposed group
			no description of the derivation of the non-exposed cohort
		Methods for determining exposure factors(1 point)	fixed file record (e.g. surgical records)★
			structured interview★
			written self-report
			no description
		Determine outcome indicators not to be observed at the start of the study (1 point)	yes ★
			no
	Comparability between groups (0-2 points)	Comparability of the cohort considered in design and statistical analysis (2 points)	study controlled for the most important confounding factors ★
			study controls for any additional factor (The criteria could be modified to indicate specific control for a second important factor.) ★
	Outcome (0-3 points)	Whether the research adequately evaluates the results (1 point)	independent blind assessment ★
			on file★
			self-report
			no description
		Results Whether follow-up was long enough after occurrence (1 point)	yes (provide appropriate follow-up time before evaluation) ★
			no

		Adequacy of follow up of cohorts (1 point)	Complete follow-up ★
			A small number of subjects were lost to follow-up without introducing bias (specifying the loss rate or describing the loss of follow-up) ★
			loss of access (set loss rate)
			follow-up was not described
Case-control sudy	Study population selection (0-4 points)	Appropriateness of case ascertainment (1 point)	yes, with independent validation★
			yes, e.g. record linkage or based on self-reports
			no description
		Representativeness of the cases (1 point)	consecutive or obviously representative series of cases★
			potential for selection biases or not stated
		Selection of Controls (1 point)	controls from the same population as the case ★
			the inpatients in the same population as the cases were used as controls
			no description
		Ascertainment of Controls (1 point)	no history of disease (endpoint)★
			no description of source
	Comparability between groups (0-2 points)	Comparability of cases and controls on the basis of the design or analysis (2 points)	study controlled for the most important confounding factors ★
			study controls for any additional factor (The criteria could be modified to indicate specific control for a second important factor.) ★
	Outcome (0-3 points)	Ascertainment of exposure (1 point)	fixed file record (e.g. surgical records) ★
			structured interviews were conducted without knowing whether the interviewees were cases or controls
			no description
		Same method of ascertainment for cases and controls (1 point)	yes ★
			no
		Non-Response Rate (1 point)	same rate for both groups ★
			no respondents described
			rate different and no designation

NOS is full of 9 points, the star is the score item.

Supplementary Table S4: Quality assessment for the cross-sectional studies using JBI.

Study	Question 1	Question 2	Question 3	Question 4	Question 5	Question 6	Question 7	Question 8	Question 9	Question 10	Total Scores
Choi et al.2018 [10]	2	2	2	2	2	1	0	2	2	2	17
Chua et al.2019 [3]	2	1	2	2	2	1	2	2	2	2	18
Yang et al.2021 [23]	2	2	1	2	2	1	0	2	2	2	16
Grant et al.2021[20]	2	2	2	2	2	1	2	2	2	2	19
Yang et al.2021 [24]	2	2	0	2	2	1	1	2	2	2	16
Chua et al.2022 [12]	2	1	2	2	1	1	2	2	2	2	17
Ju et al.2022 [25]	2	1	2	2	2	1	0	2	2	2	16

Supplementary Table S5: Quality assessment for the cohort studies and case-control studies using NOS.

References	Quality assessment (NOS)			Total score [1]
	Selection	Comparability	Outcome	
Chen et al.2022 [13]	4	1	3	8
Chang et al.2019 [5]	2	1	3	6
Shin et al.2020 [21]	4	2	3	9
Sun et al.2021 [22]	3	1	2	6
Li et al.2022 [26]	3	1	3	7

[1] The score ranged from 0 to 9 (selection<=4, comparability<=2, outcome<=3). Low-quality research, scored 0–3; moderate quality research, scored 4–6; high-quality research, scored 7–9.

Supplementary Table S6: Egger's test for publication bias of studies exploring exposure to different pollutants and vision disorder.

Pollutant	Original Studies (N)	*P*
**PM_{10}**	6	0.512
**$PM_{2.5}$**	10	0.049
**SO_2**	4	0.016
**NO_2**	6	0.488
**O_3**	3	0.051

Abbreviations: $PM_{2.5}$: particle with aerodynamic diameter ≤ 2.5 μm; PM_{10}: particle with aerodynamic diameter ≤ 10 μm; NO_2: nitrogen dioxide; SO_2: sulfur dioxide; O_3:ozone; CO: carbon monoxide; OR: odds ratio; CI: confidence interval.

* p-value < 0.05.

Supplementary Figure S1: Sensitivity analysis results of PM_{10}.

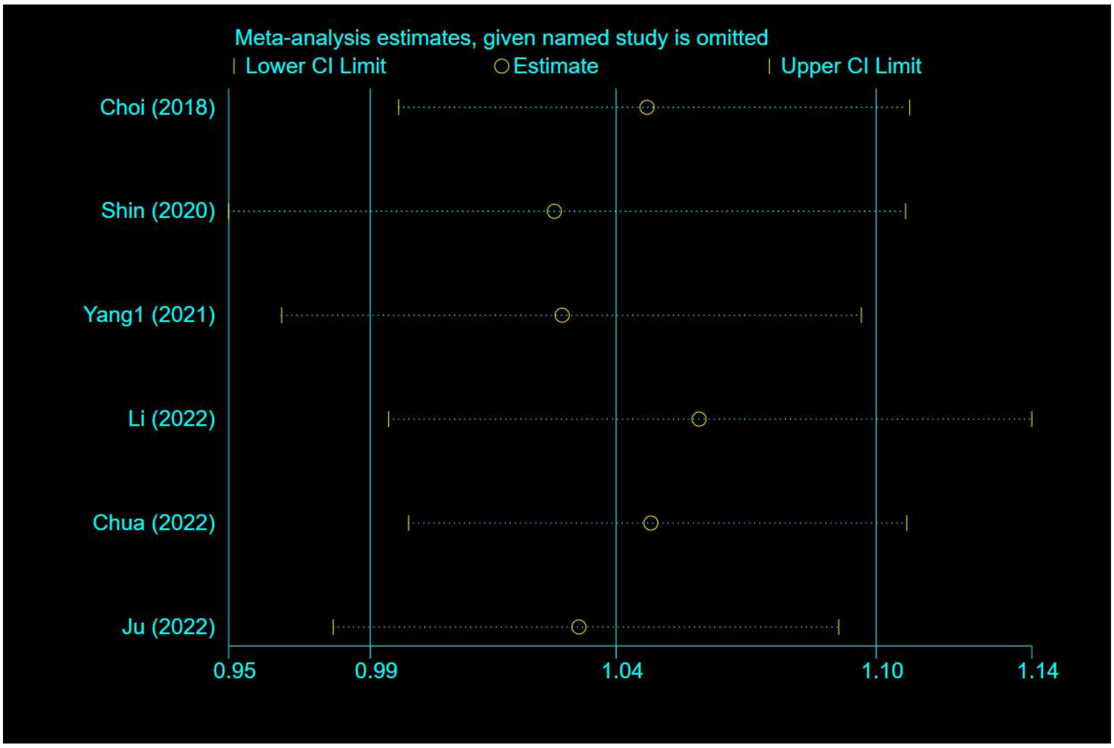

Supplementary Figure S2: Sensitivity analysis results of $PM_{2.5}$.

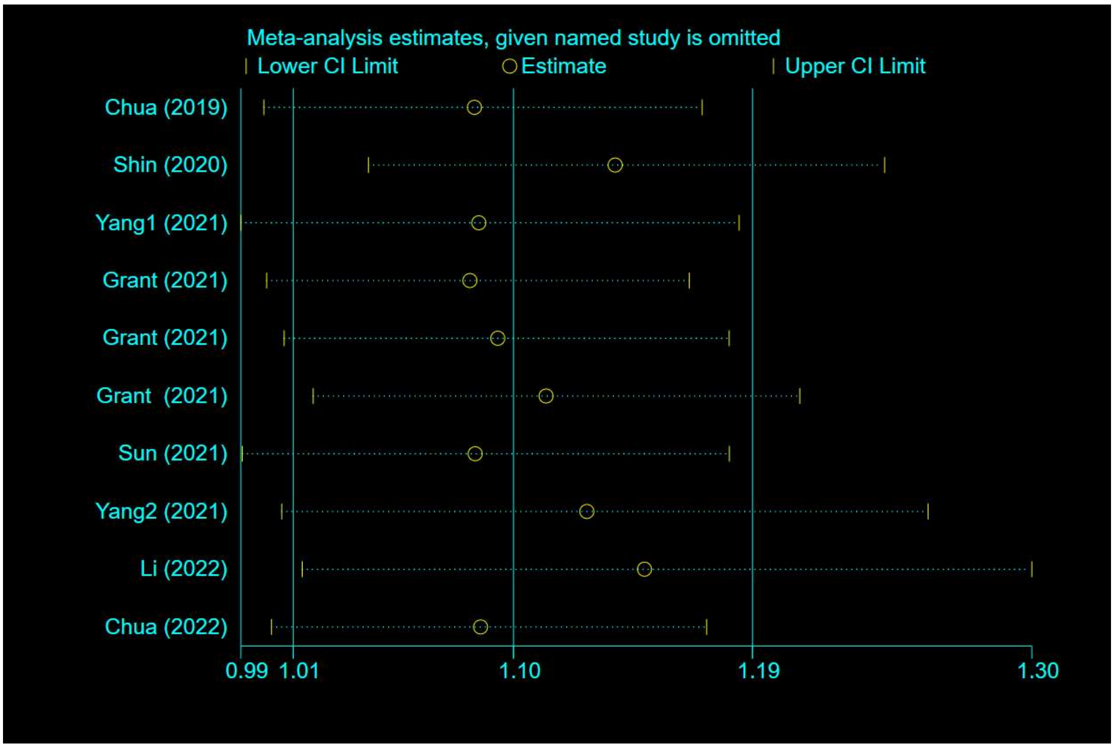

Supplementary Figure S3: Sensitivity analysis results of SO_2.

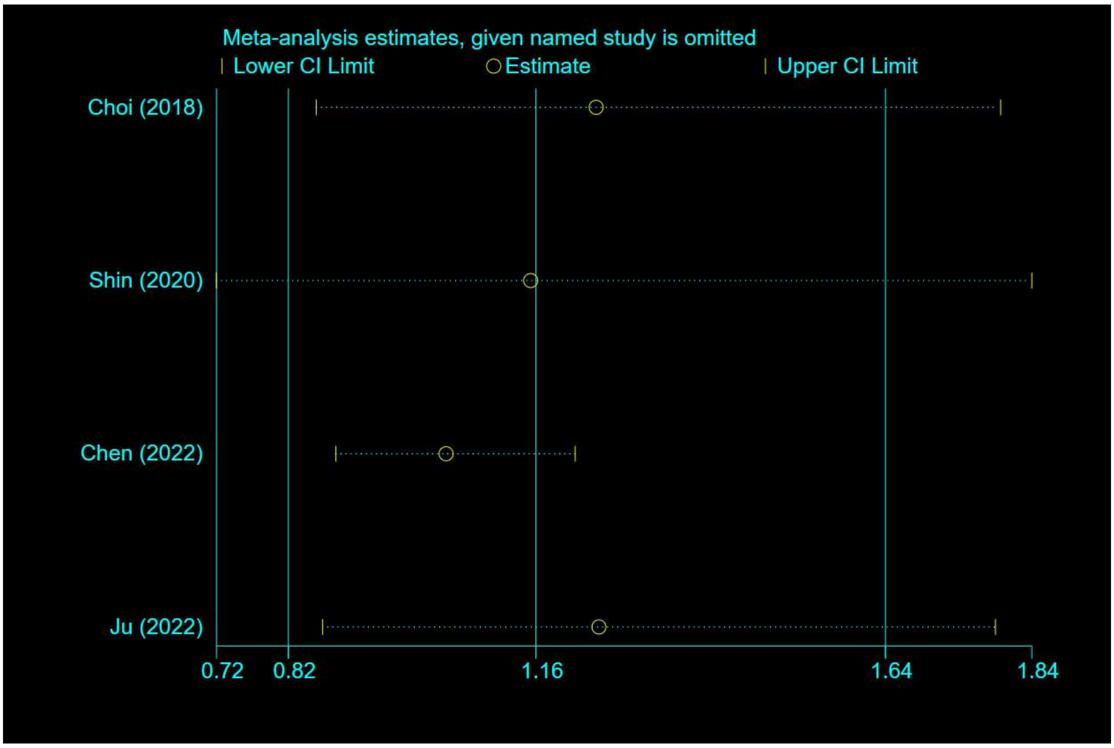

Supplementary Figure S4: Sensitivity analysis results of NO_2.

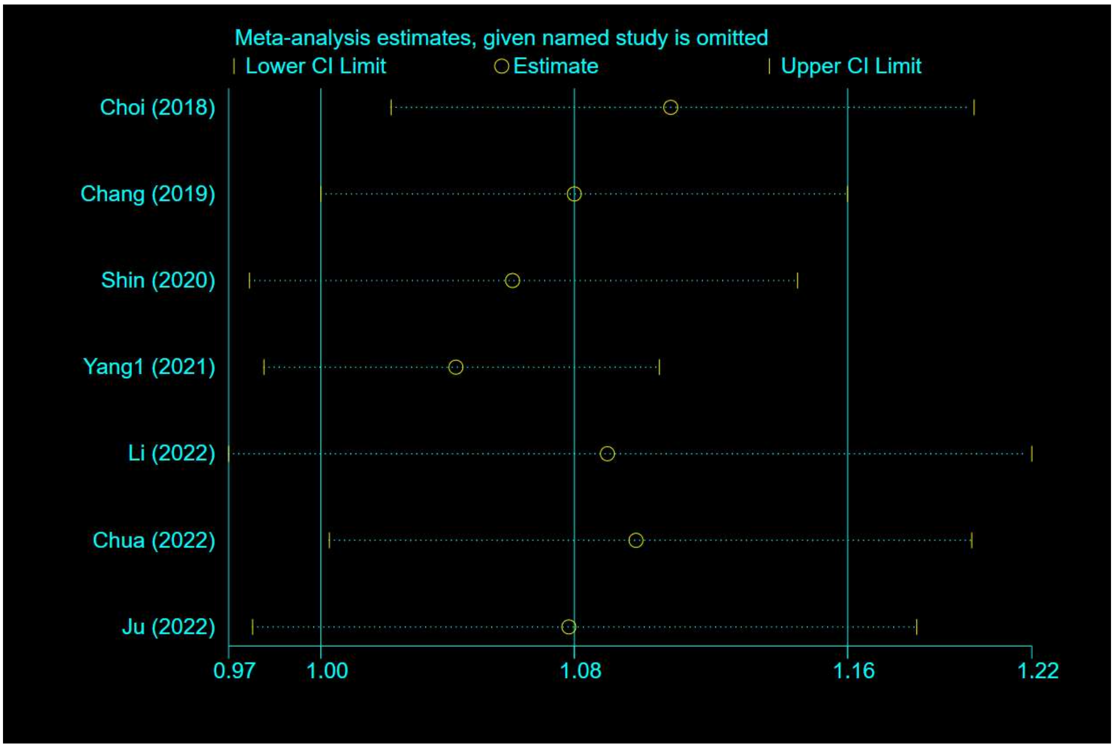

Supplementary Figure S5: Sensitivity analysis results of O_3.

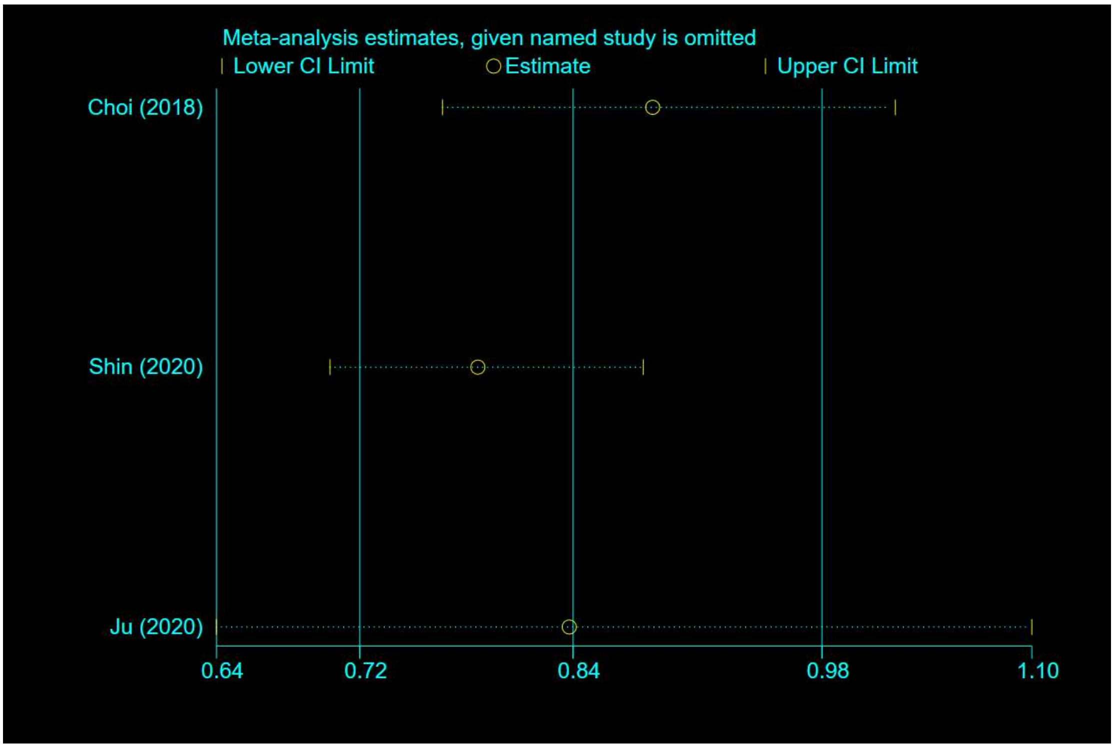

Supplementary Figure S6: Sensitivity analysis results of CO.

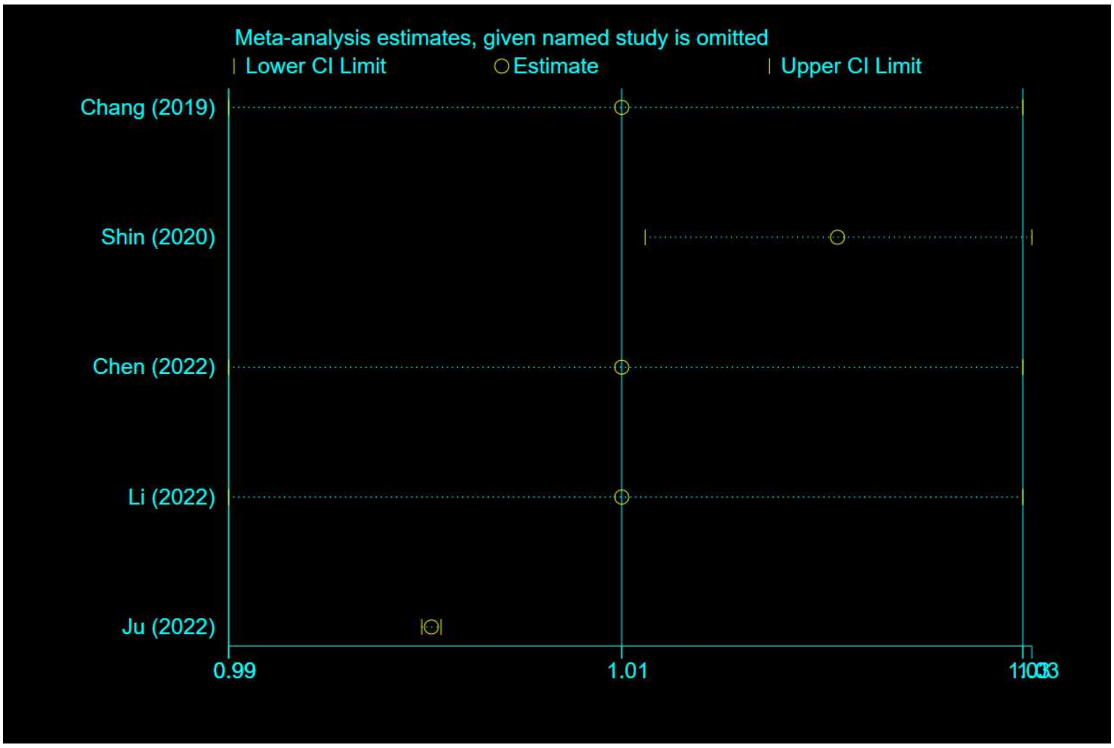

Supplementary Figure S7: Funnel plot for publication bias of studies exploring exposure to PM_{10} and vision disorder.

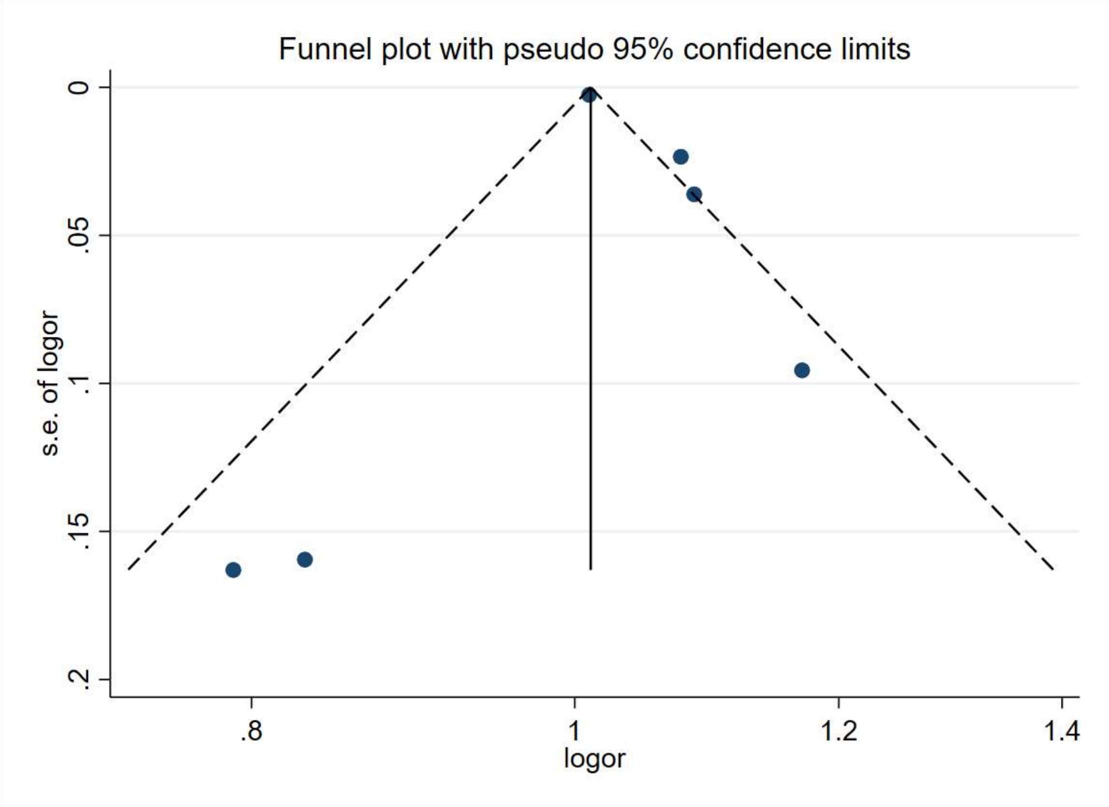

Supplementary Figure S8: Funnel plot for publication bias of studies exploring exposure to $PM_{2.5}$ and vision disorder.

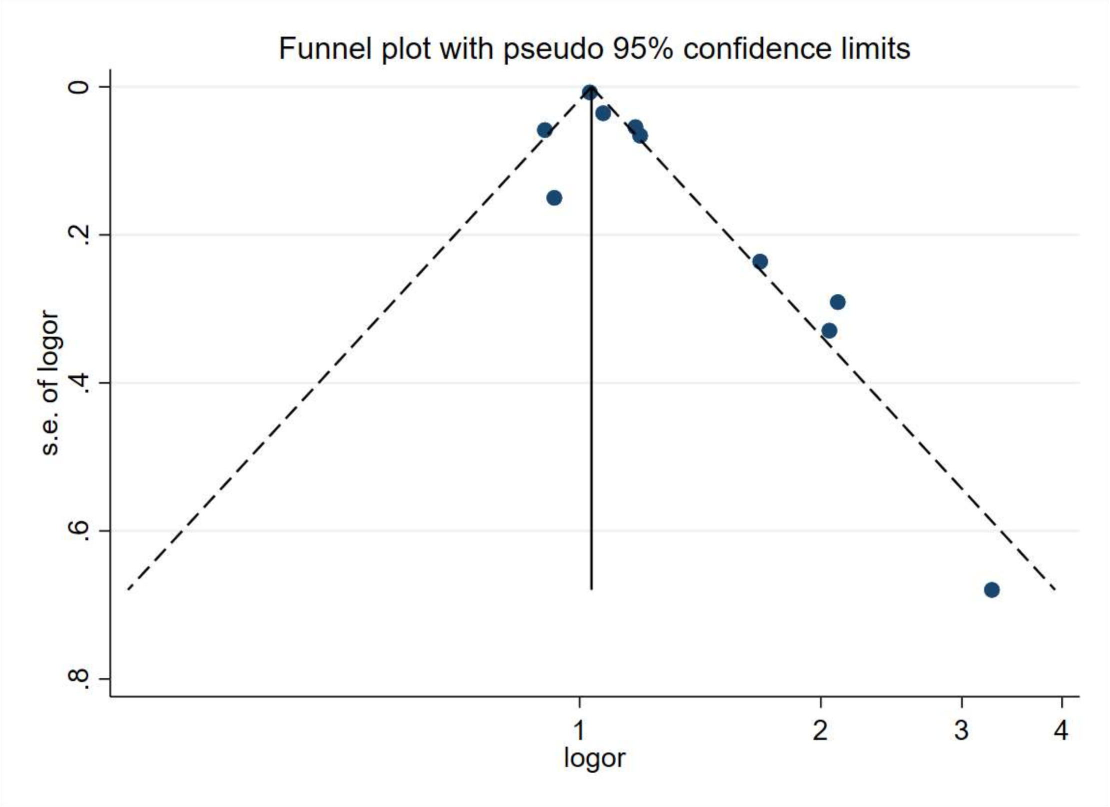

Supplementary Figure S9: Funnel plot for publication bias of studies exploring exposure to SO_2 and vision disorder.

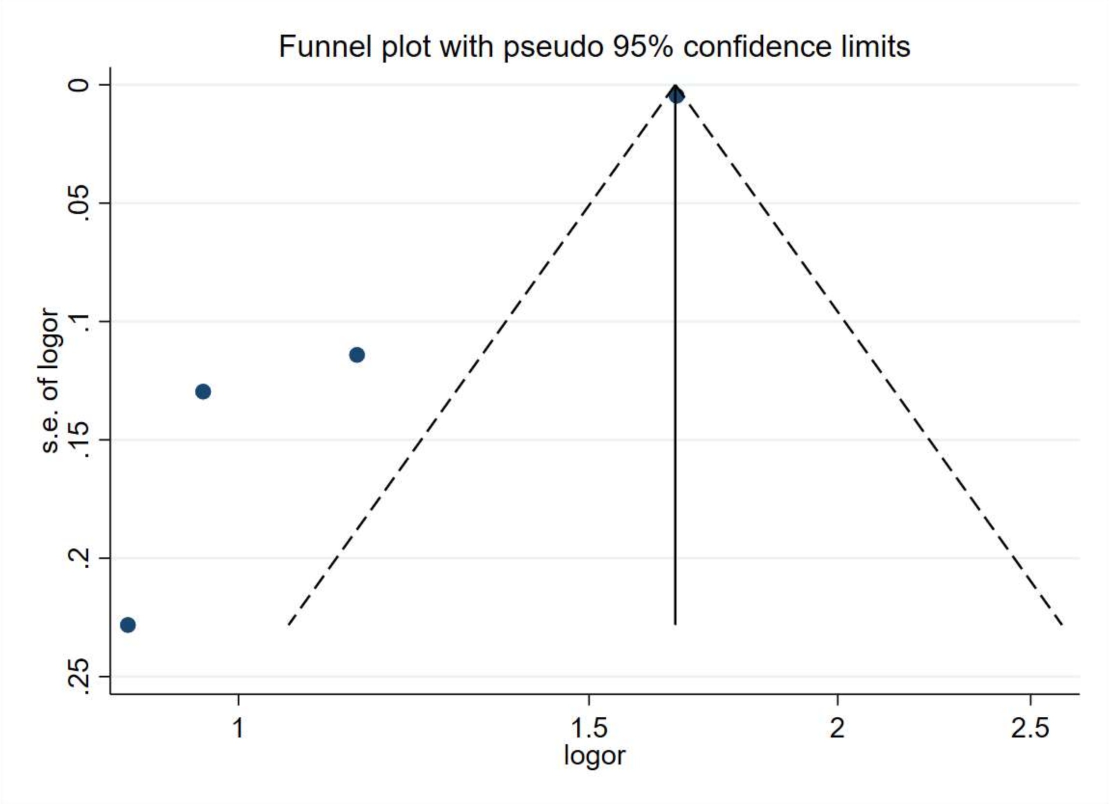

Supplementary Figure S10: Funnel plot for publication bias of studies exploring exposure to NO_2 and vision disorder.

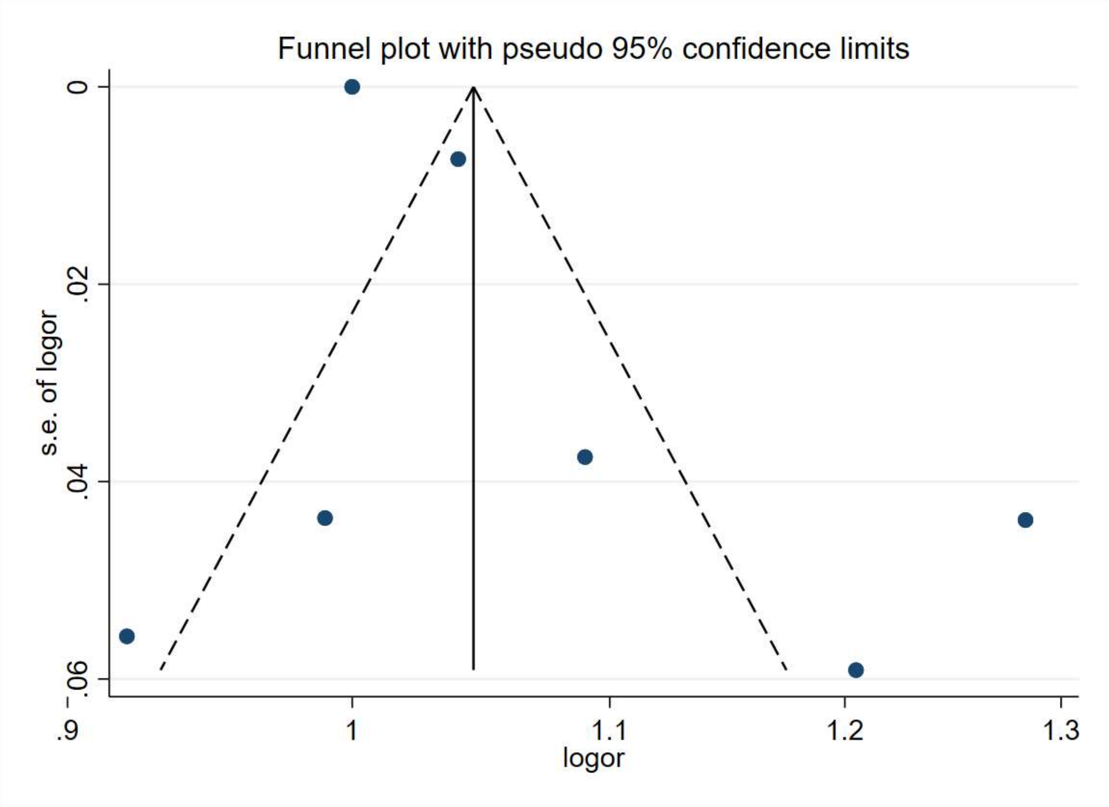

Supplementary Figure S11: Funnel plot for publication bias of studies exploring exposure to O_3 and vision disorder.

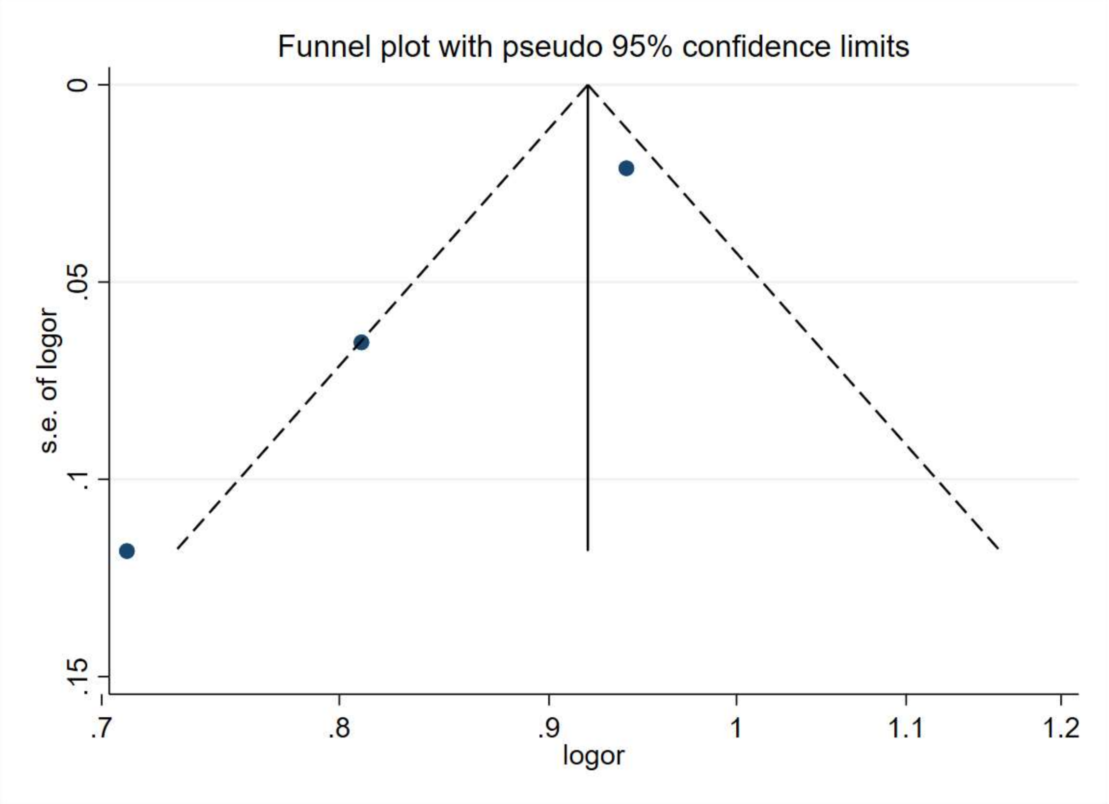

Supplementary Figure S12: Funnel plot for publication bias of studies exploring exposure to CO and vision disorder.

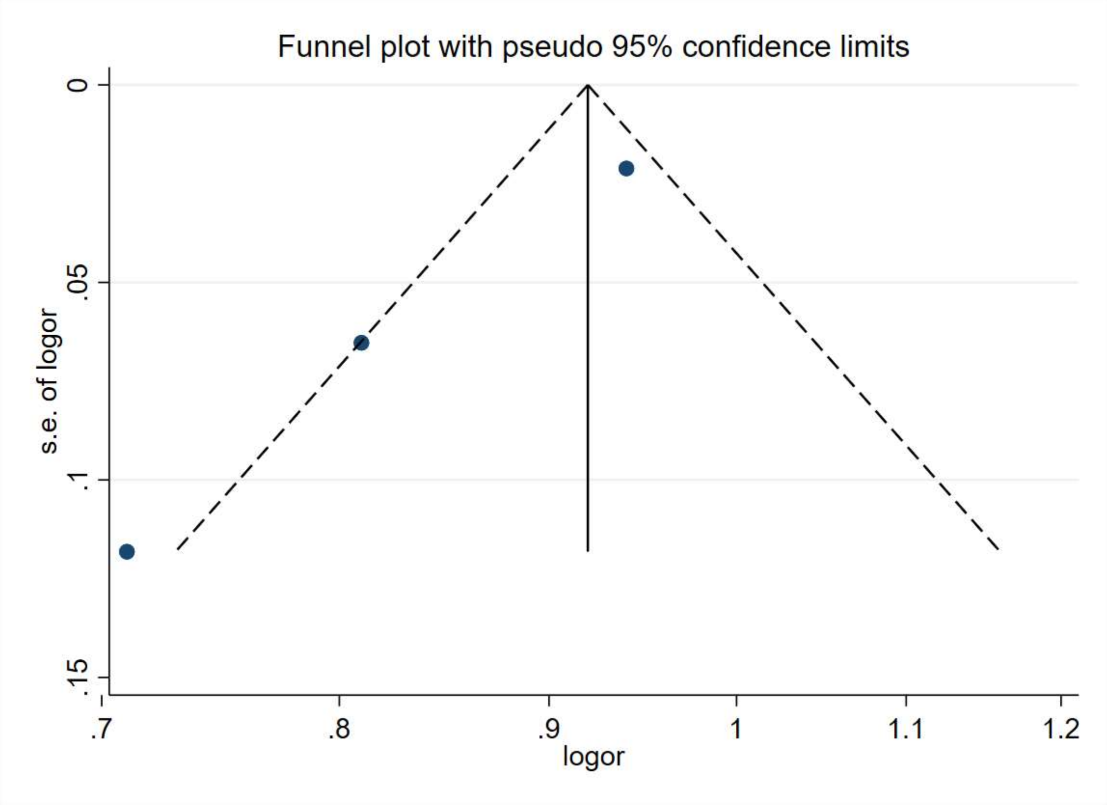

Funnel plot with pseudo 95% confidence limits
s.e. of logor
0
.05
.1
.15
.7
.8
.9
1
1.1
1.2
logor
